# Mechanistic insights into the formation and spread of carbapenem-resistant hypervirulent Klebsiella pneumoniae via an IncHI1B/repB hybrid virulence plasmid

**DOI:** 10.1099/mgen.0.001534

**Published:** 2025-10-24

**Authors:** Binghui Huo, Yuxuan Liu, LinPing Fan, LiFan Xu, WenJing Yang, DanDan Wei, Peng Liu, Shanshan Huang, HanXu Hong, Yang Liu

**Affiliations:** 1 Department of Clinical Laboratory, The First Affiliated Hospital, Jiangxi Medical College, Nanchang University, 17 Yongwai Zhengjie, Nanchang 330006, PR China; 2 Department of Clinical Laboratory, Peking University People’s Hospital, Beijing, PR China; 3 School of Public Health, Jiangxi Medical College, Nanchang University, Nanchang, Bayi Avenue No. 461, 330006, PR China; 4 The First Clinical Medical College of Nanchang University, Nanchang, Jiangxi, PR China; 5 China-Japan Friendship Jiangxi Hospital, National Regional Center for Respiratory Medicine, Nanchang city 330200 Jiangxi, PR China; 6 Jiangxi Medical Center for Critical Public Health Events, The First Affiliated Hospital, Jiangxi Medical College, Nanchang University, Nanchang 330052, PR China

**Keywords:** *blaKPC*–*rmpA2*, fusion plasmid, horizontal transfer, *Klebsiella pneumoniae*, mobile genetic element (MGE)

## Abstract

**Objectives.** The transfer of virulence plasmids facilitates the emergence of carbapenem-resistant hypervirulent *Klebsiella pneumoniae* (CR-hvKP), complicating infection management. This study identifies a conjugative IncHI1B/repB hybrid virulence plasmid (pHvKP51-Vir) that promotes CR-hvKP formation via recombination within a highly active mobile genetic element (MGE) region during conjugative transfer.

**Methods.** The fusion of the KPC-type resistance plasmid (pKPC) with the virulence plasmid (pHvKP51-Vir) was demonstrated via conjugation experiments, leading to the formation of the hybrid plasmid pJ53-KPC_Vir. Plasmid conjugative transfer experiments, Southern blotting, *Galleria mellonella* infection assays and serum killing assays were utilized to investigate alterations in pHvKP51-Vir and its fusion with pKPC during conjugation.

**Results.** The fusion plasmid pJ53-KPC_Vir co-harboured *bla_KPC_* and *rmpA2*, conferring both carbapenem resistance and virulence. The fusion plasmid demonstrated high transferability, facilitating the dissemination of virulence plasmids. While the fusion plasmid temporarily attenuated virulence, its dissociation restored the virulence plasmid to its original level. TnAs1 and the MGE region were critical in mediating this fusion.

**Conclusions.** The TnAs1- and MGE-mediated fusion generated an unstable hybrid plasmid with high transferability. Its dissociation restored and enhanced virulence, promoting rapid dissemination of resistance and virulence, posing a substantial clinical threat.

**Impact Statement**

The emergence of carbapenem-resistant hypervirulent *Klebsiella pneumoniae* (CR-hvKP) poses a significant clinical challenge, as it combines both antibiotic resistance and virulence, complicating treatment strategies. This study identifies a novel conjugative hybrid plasmid, pJ53-KPC_Vir, formed through the fusion of a KPC-type resistance plasmid and a virulence plasmid, mediated by mobile genetic elements (MGEs). The fusion plasmid exhibits high transferability, facilitating the rapid spread of both resistance and virulence traits. While the hybrid plasmid temporarily attenuates virulence, its dissociation restores and enhances virulence, thus promoting the dissemination of CR-hvKP. These findings highlight the dynamic nature of plasmid recombination and the potential for rapid emergence of highly virulent, resistant strains, which pose a significant threat to public health. Understanding the mechanisms behind plasmid fusion and transfer may guide future strategies to control the spread of CR-hvKP.

## Data Summary

The complete genome sequences of pHvKP51-Vir (accession number: CP157321), pJ53-KPC_Vir (accession number: CP157326), pKPC (accession number: CP157324) and pJ53-KPC_Vir-P15 (accession number: CP158675) are deposited in GenBank of the National Center for Biotechnology Information. The accession numbers for all other genome sequences analysed in this study are as follows: pHSKP107-2 (accession number: CP100108.1), pINF242-SC-2 (accession number: LR890625.1), pKP8-2 (accession number: CP025638.1), J53 (accession number: PRJNA82837) and ATCC700603 (accession number: SAMEA5402511).

## Background

The global rise of *Klebsiella pneumoniae* strains that simultaneously exhibit high levels of antimicrobial resistance and enhanced virulence – referred to as carbapenem-resistant hypervirulent *K. pneumoniae* (CR-hvKP) – poses an escalating threat to public health and clinical therapeutics [1,6]. These strains represent a convergence of two historically distinct evolutionary trajectories: the acquisition of carbapenemase-encoding resistance genes, such as *bla*_KPC_, *bla*_NDM_ and *bla*_OXA-48_-like, and the presence of classical virulence loci including *rmpA*, *rmpA2* and siderophore systems (*iuc, iro*) that are typically found on large virulence plasmids [7,9]. Whole-genome sequencing and virulence phenotyping have demonstrated that CR-hvKP isolates can maintain a high virulence potential even after acquiring two or more carbapenemase genes [10]. CR-hvKP infections are frequently associated with invasive disease, rapid clinical deterioration and high mortality, particularly in immunocompromised patients or those in intensive care units [11,12].

The emergence of CR-hvKP is increasingly attributed to the horizontal gene transfer of plasmids that co-harbour both resistance and virulence determinants, giving rise to resistance–virulence hybrid plasmids [13,15]. These chimeric plasmids are often the result of recombination events between classical resistance plasmids and mobilizable virulence plasmids, mediated by mobile genetic elements (MGEs) such as insertion sequences (e.g. IS26, IS1), transposons and integrative conjugative elements. Plasmids carrying multiple carbapenemase genes can undergo IS26-mediated recombination, rearrangement or fusion, enabling their horizontal dissemination and contributing to the increasingly complex resistance profiles of CR-hvKP [16,17].

Although traditionally considered to have low transfer frequency and underestimated transmission risk [18,21], recent studies have revealed that even virulence plasmids lacking complete conjugation machinery can be mobilized through co-transfer or fusion with helper plasmids, thus facilitating their dissemination [22]. MGEs, including insertion sequences such as IS26 and conjugative transposons, play a pivotal role in promoting plasmid recombination and gene transfer [15,23]. The conjugative ability of plasmids depends on the origin of transfer (oriT) and associated transfer genes (*tra/ trb*). While some virulence plasmids lack oriT, they can acquire transferability by fusing with conjugative resistance plasmids. Moreover, mobilizable plasmids, which lack a complete conjugation system, have also been shown to transfer effectively with the assistance of helper plasmids, further accelerating the dissemination of both resistance and virulence determinants [24].

Notably, genomic surveillance has revealed an increasing number of CR-hvKP isolates carrying large hybrid plasmids formed by the fusion of pK2044-like virulence plasmids with resistance plasmid backbones belonging to IncFII, IncHI1B or IncFIB incompatibility groups. These chimeric plasmids are prevalent among epidemic strains such as ST11-KL64 in China and diverse clinical clones including ST15, ST147 and ST23 in Japan and Southeast Asia. They frequently harbour key resistance genes such as *bla*_KPC_ alongside virulence genes including *iuc*ABCD and *rmpA2* [25,27]. For instance, in ST11-KL64 strains circulating in China, representative hybrid plasmids such as pKP1838-KPC-vir and pKP18-2079-vir have been identified, resulting from recombination between classical virulence plasmids and multidrug-resistant plasmids [28]. Furthermore, the co-existence of virulence and resistance plasmids is commonly observed in clinical isolates, with stable inheritance maintained through plasmid co-maintenance mechanisms, facilitating their long-term persistence and widespread dissemination [29].

Despite these advances, the molecular basis of plasmid fusion events remains insufficiently characterized. Critical aspects such as the features of recombination hotspots, the roles of site-specific recombinases or transposases and the structural constraints of involved plasmids have yet to be fully elucidated. A deeper understanding of these mechanisms is essential for clarifying the emergence and dissemination dynamics of CR-hvKP and for developing effective strategies for its containment and clinical management.

## Methods

### Bacterial strains

The clinical samples employed in this study were obtained from the First Affiliated Hospital of Nanchang University, Jiangxi Province, China. For the conjugation assays, *Escherichia coli* J53 and *K. pneumoniae* ATCC700603 were used as receptor bacteria [30].

### Conjugation assay

HvKP51 harboured a virulence plasmid resistant to potassium tellurite. The transferability of pHvKP51-Vir and pKPC was assessed via conjugation assays in lysogeny broth (LB) using azide-resistant *E. coli* J53 as recipients. After overnight incubation, donor and recipient strains were cultured at 200 r.p.m. and 37 °C to the logarithmic growth phase (OD_600_=1) in LB medium. A 4-h conjugation assay minimizes the likelihood of secondary transfer events from transconjugants to recipient cells, although this possibility cannot be completely excluded. Moreover, limiting the mating duration to 4 h helps mitigate the influence of potential growth rate differences among donors, recipients and transconjugants on the accurate assessment of conjugation frequency. One millilitre of donor and recipient cells was washed with PBS, resuspended in 20 µl of 10 mM MgSO4, combined and inoculated onto LB agar plates. Following overnight incubation at 37 °C, the bacteria were resuspended, serially diluted in PBS and spread onto antibiotic-supplemented LB agar plates for transconjugant selection. Antibiotics and their respective concentrations for each conjugate pair are detailed in Fig. 2. Each experiment was performed in triplicate to ensure reliability of horizontal transfer frequencies. Transconjugants were validated using *XbaI* and S1 nuclease PFGE coupled with PCR. Conjugation frequency was determined as the ratio of transconjugants to recipients based on c.f.u. counts from serially diluted antibiotic-containing plates [14,31]. The primers utilized for the PCR amplification of the *bla_KPC_* gene were as follows: forward primer 5′-GCTACACCTAGCTCCACCTTC-3′ and reverse primer 5′-ACAGTGGTTGGTAATCCATGC-3′. Similarly, the primers designed for amplifying the *rmpA2* gene were forward primer 5′-GGATGTGGCTTGACATTTCGGGGG-3′ and reverse primer 5′-TTCATGGATGCCCTCCCTCCTG-3′.

### Whole-genome sequencing and annotation

Genomic DNA from strains HvKP51, HvKP51-KPC-TC, J53-KPC_Vir-TC and J53-KPC_Vir-P15 was extracted using the QIAamp DNA Mini Kit (QIAGEN, Germany). For PacBio RS sequencing, 5 µg of genomic DNA was fragmented using g-TUBE (Covaris, USA), and a 10 kb library was constructed following PacBio Sequel2 sample preparation guidelines. Sequencing was performed on the PacBio RS II platform (Menlo Park, CA). Additionally, a 300-bp paired-end library was prepared according to Illumina TruSeq DNA sample preparation protocols and sequenced on the HiSeq 2500 platform with a read length of 150 bp.

PacBio sequencing data were assembled using the Hierarchical Genome Assembly Process, generating a single-contig assembly. High-coverage (~300×) Illumina data were aligned to the PacBio assembly using Bowtie2 and Samtools for error correction. The final assembly yielded a complete, non-redundant genome.

Gene predictions were subjected to blast analysis against the non-redundant database on National Center for Biotechnology Information (NCBI) for functional annotation. Plasmid replicon typing was performed using the PlasmidFinder tool (PlasmidFinder 2.1 (dtu.dk)) [32]. Conjugative transfer-related modules, including the relaxase gene, type IV coupling protein (T4CP) gene and the tra gene cluster for the type IV secretion system (T4SS), were identified using oriTfinder [oriTfinder (sjtu.edu.cn)] (Table S1, available in the online Supplementary Material) [33]. Mobile genetic elements such as I*S903*, *IS110* and *IS407* were detected using ISfinder [ISfinder (biotoul.fr)] [34].

### Plasmid stability in transconjugants

To determine plasmid stability, a single colony from each transconjugant was picked from freshly streaked agar plates and cultured in LB broth. The transconjugants were serially cultured for 2 weeks, with 5 µl of bacterial suspension transferred to 5 ml of fresh LB broth every 16 h. Plasmid stability was assessed daily by comparing the number of colonies on antibiotic-free plates with those on plates containing 2 µg ml^−1^ meropenem and 5 µg ml^−1^ dipotassium trioxotellurate. Three colonies were randomly selected each day for antibiotic resistance testing and PCR detection of the *rmpA2* gene from the virulence plasmid and the *bla_KPC_* gene from the resistance plasmid. S1 nuclease-PFGE analysis was conducted on strains positive for both *bla_KPC_* and *rmpA2* [14].

### S1-PFGE and Southern blotting hybridization

Plasmid characteristics were assessed using S1-PFGE. To determine the localization of the *rmpA2* gene on the virulence plasmid and the *bla_KPC_* gene on the resistance plasmid, Southern blot hybridization was performed. Following previously described methods, the separated plasmids were transferred to Hybond-N^+^ membranes (Amersham) [35]. Probes for *rmpA2/bla_KPC_* labelling and hybridization were performed with the DIG-High Prime DNA Labelling and Detection Starter Kit I (cat. no. 11745832910, Roche, Mannheim, Germany). The hybridization temperatures for the primers used in the Southern blot analysis were set as follows: 50 °C for *rmpA2* primers and 65 °C for *bla_KPC_* primers.

### *Galleria mellonella* larval infection assay and the serum killing assay

The assessment of virulence potential was carried out utilizing the well-established G. mellonella wax moth *in vivo* infection model [36,37] and the serum killing assay [38]. Bacterial cultures were prepared overnight, rinsed and adjusted to 10^8^ c.f.u. ml^−1^ in PBS. Ten larvae per group were injected with 10 µl of the bacterial suspension, while control larvae received PBS. The infected larvae were incubated in sterilized Petri dishes at 37 °C for 60 h, with survival rates recorded every 12 h. Each experiment was repeated three times. Results were analysed using GraphPad Prism 9.5.1. Differences in survival rates were assessed using the log-rank (Mantel-Cox) test. *P<0.05; ***P<0.001. Serum resistance was assessed following the procedure described by Podschun *et al*. [38]. In brief, 25 µl of a mid-log phase culture, corresponding to 2.5×10⁶ c.f.u. ml^−1^, was mixed with 75 µl of normal human serum obtained from healthy volunteers. The mixture was incubated at 37 °C for 1, 2 and 3 h. Following incubation, the samples were diluted and plated on LB agar for colony counting. Results were evaluated as per previous studies, and all assays were performed in triplicate. Results were analysed using GraphPad Prism 9.5.1.

### Statistical analysis

Statistical analysis was performed using SPSS 26 software. The data are presented as the means±sd based on three independent experiments. Survival rate analyses were conducted using SPSS 26 and GraphPad Prism (9.5.1). Results with *P*<0.05 in a two-tailed test were considered statistically significant.

## Results

### Whole-genome sequencing analysis revealed a recombination region – MGEs – in a novel conjugative virulence plasmid pHvKP51-Vir

**Fig. 1.**
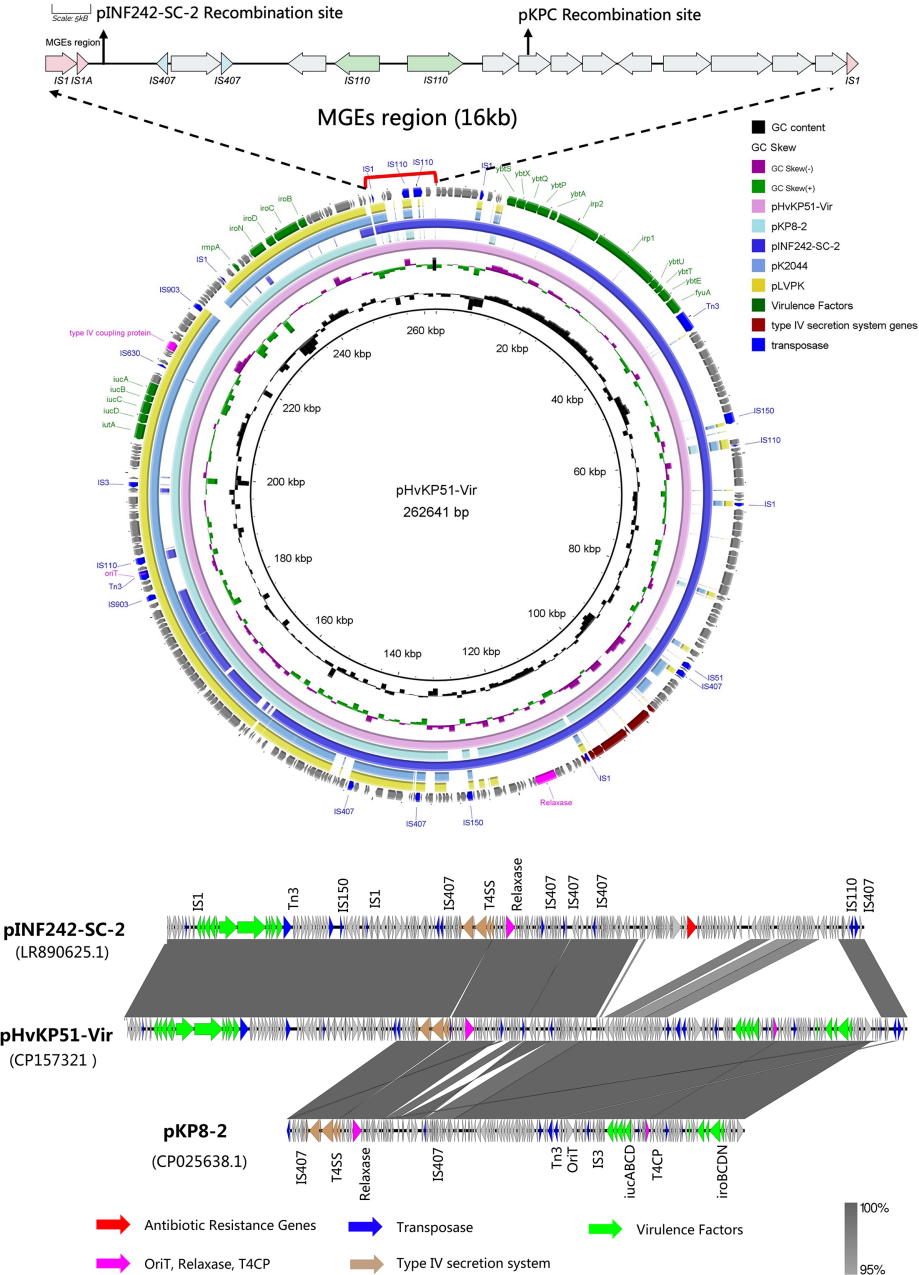
Whole-genome sequencing analysis of fusion-promoting factors in pHvKP51-Vir. The hybrid plasmid pHvKP51-Vir is a recombinant formed from pKP8-2 and pINF242-SC-2. The recombination region is enriched with MGEs containing numerous conjugative transfer elements. Whole-genome sequencing identified two recombination events within the MGEs region of the hybrid plasmid pHvKP51-Vir.

We isolated a hypervirulent strain of *K. pneumoniae* (HvKP51) obtained from a patient with a severe clinical infection. Whole-genome sequencing revealed that HvKP51 belongs to the ST485/KL5 lineage of *K. pneumoniae*. Its virulence plasmid was designated as pHvKP51-Vir (Fig. 1). This plasmid harbours several critical virulence genes, including *rmpA*/*rmpA2*, which regulate the mucous phenotype, *iucABCD*, responsible for aerobactin synthesis, and *iroBCDN*. These genes confer a hypermucoviscous phenotype. Infection experiments using *G. mellonella* larvae confirmed the high virulence of this strain. Notably, long-read whole-genome sequencing and plasmid replicon analysis (PlasmidFinder) confirmed that pHvKP51-Vir is the only plasmid present in HvKP51, and it harbours an IncHI1B/repB replicon. No additional plasmids were detected. Whole-genome sequencing analysis revealed that pHvKP51-Vir carries a recombination-facilitating region enriched with numerous insertion sequences (IS) *–* the MGEs region.

Whole-genome analysis revealed that pHvKP51-Vir was formed by the recombination of two plasmids: pKP8-2 (99.99% identity, 92% coverage, totaling 143,362 bp) and pINF242-SC-2 (99.93% identity, 100% coverage, totaling 107,512 bp). A 10 kb region at the junction of pKP8-2 and pINF242-SC-2, enriched with insertion sequences, is referred to as the MGE region in this study. The sequence within the MGEs region is IS1-pINF242-2 insertion site-IS407-IS407-IS110-IS110-IS1. The IS1 element is crucial for the recombination of pHvKP51-Vir, promoting homologous recombination between the pKP8-2 and the pINF242-SC-2 (Fig. 1).

### Fusion plasmid formation via conjugative transfer facilitates CR-hvKP emergence and dissemination

**Fig. 2.**
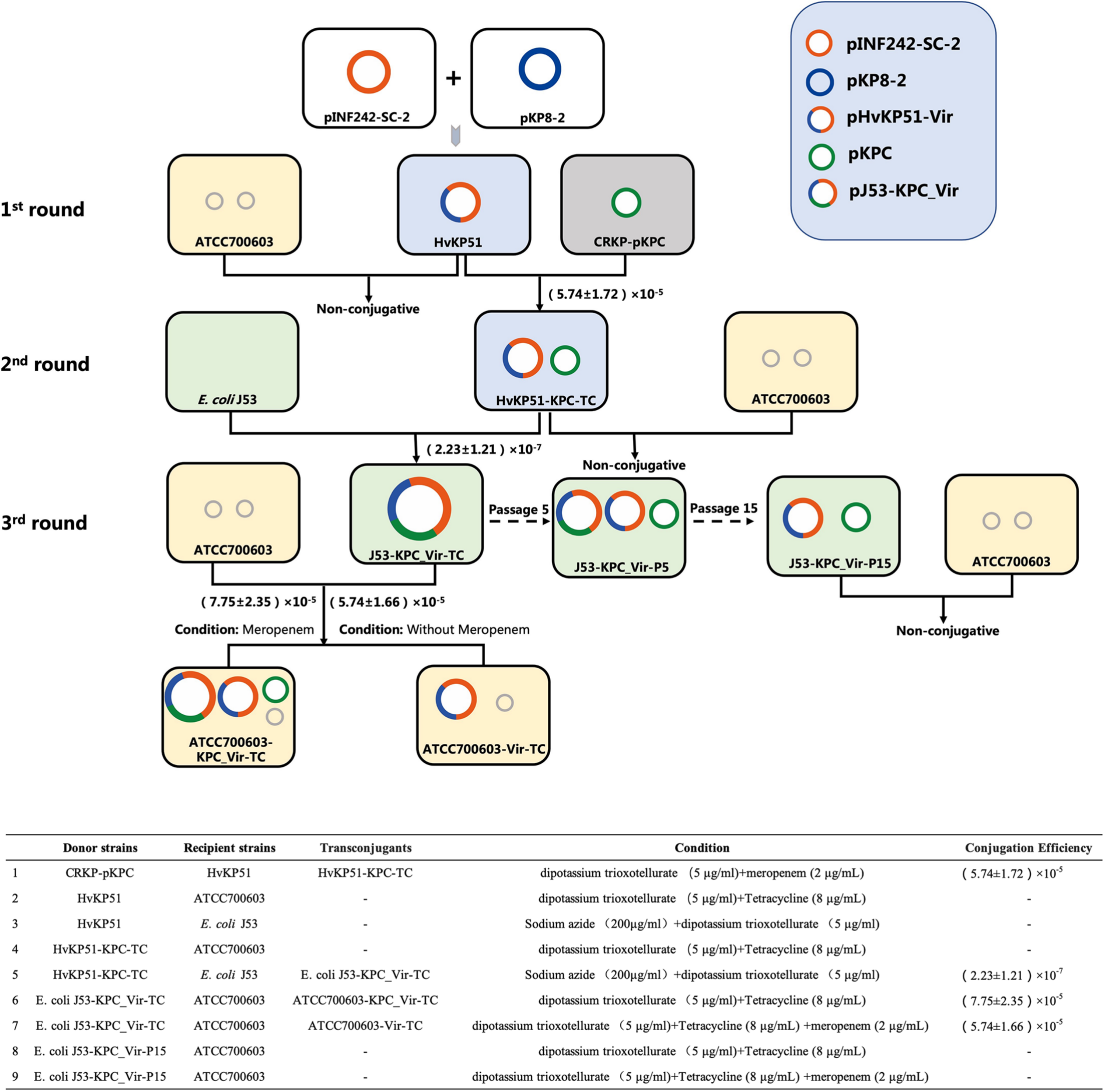
Horizontal conjugative transfer of the fusion plasmid pJ53-KPC_Vir, formed by the hybrid plasmid pHvKP51-Vir fusing with pKPC, in *K. pneumoniae* ATCC700603 and *E. coli* J53. In the first round of conjugation experiments, HvKP51 was used as the recipient bacterium, which resulted in the transconjugant HvKP51-KPC-TC. In the second round, HvKP51-KPC-TC was used as the donor bacterium and *E. coli* J53 as the recipient bacterium, which resulted in the transconjugant J53-KPC_Vir-TC. In the third round, J53-KPC_Vir-TC was used as the donor bacterium, and *K. pneumoniae* ATCC700603 as the recipient bacterium, which resulted in the transconjugants ATCC700603-KPC_Vir-TC and ATCC700603-Vir-TC.

**Fig. 3.**
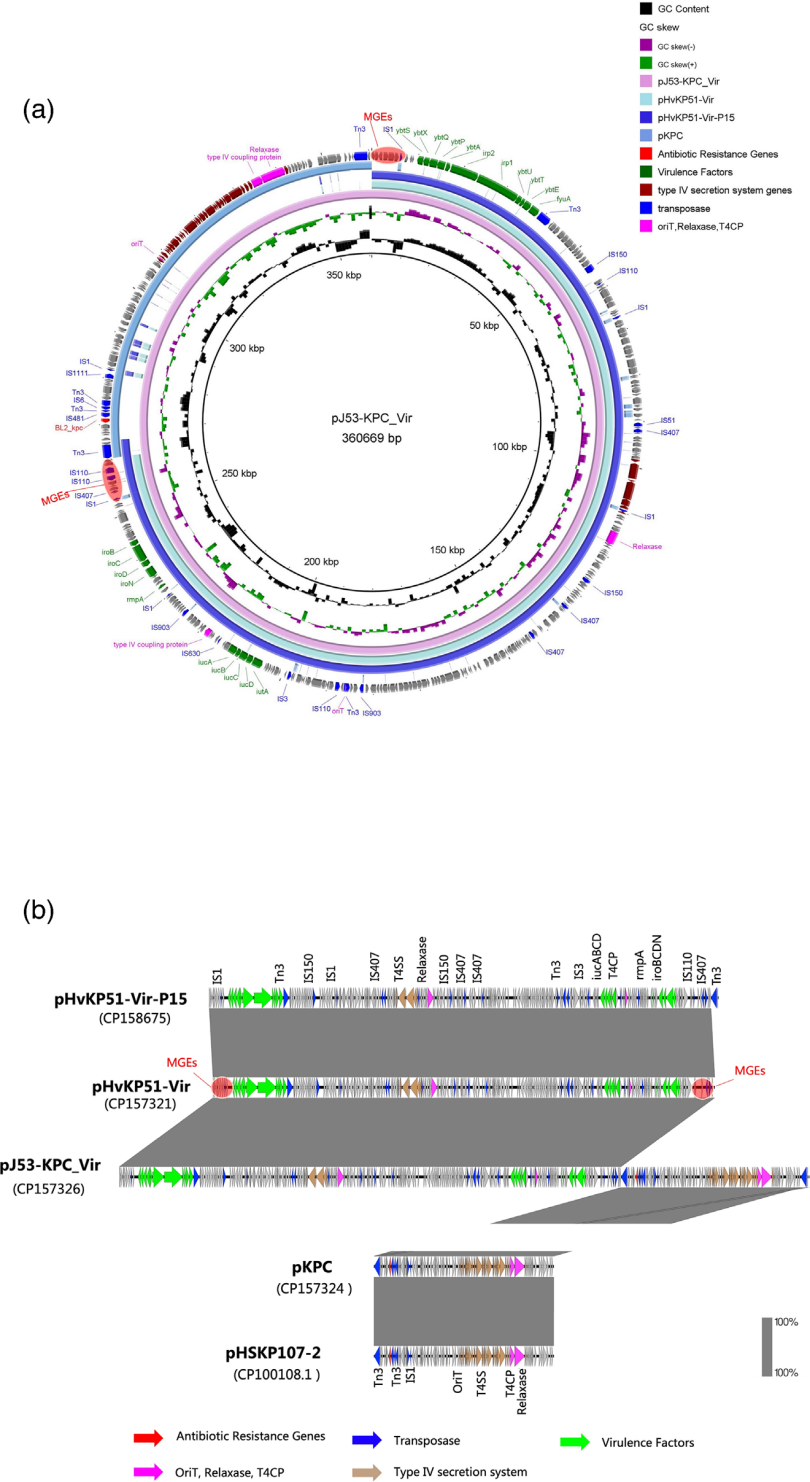
Plasmid sequencing analysis of the fusion plasmid pJ53-KPC_Vir. (a, b) The fusion plasmid pJ53-KPC_Vir was identified as a recombinant plasmid formed through the fusion of pHvKP51-Vir and pKPC, occurring within the MGEs region of pHvKP51-Vir and the TnAs1 element of pKPC. After 15 days of serial passage, pJ53-KPC_Vir underwent dissociation at the original fusion site, yielding the virulence plasmid pHvKP51-Vir-P15. Plasmid sequencing analysis revealed that pHvKP51-Vir-P15 remained identical to the pre-dissociation plasmid pHvKP51-Vir, with the only notable change being the presence of an additional TnAs1 element in the MGEs region.

To evaluate the impact of this hybrid plasmid on the dissemination of virulence plasmids in *K. pneumoniae* and *E. coli* and to understand the conjugative transfer mechanisms, we conducted a series of conjugative transfer experiments with HvKP51 using *E. coli* J53 and *K. pneumoniae* ATCC700603 as recipients (Fig. 2). Although in *vitro* conjugation experiments did not directly observe the transfer of virulence plasmids, they demonstrated that the pKPC plasmid efficiently transferred into HvKP51 with a conjugation efficiency of (5.74±1.72) × 10^−5^. Subsequently, during conjugation with *E. coli* J53, the pKPC plasmid recombined with the virulence plasmid pHvKP51-Vir to form a new plasmid, with a conjugation efficiency of (2.23±1.21) × 10^−7^. The transconjugant J53-KPC_VIR-TC harboured a new plasmid ~360 kb in size, confirmed by S1-PFGE and Southern blot analysis to carry both the *rmpA2* and *bla_KPC_* genes. Whole-genome sequencing validated the formation of the carbapenem resistance-encoding and virulence-encoding plasmid through the fusion of pHvKP51-Vir and pKPC, designated as pJ53-KPC_Vir (Fig. 3). The newly formed fusion plasmid pJ53-KPC_Vir exhibits high transmission efficiency between *K. pneumoniae* and *E. coli*, significantly enhancing the dissemination of virulence plasmids. The transconjugant J53-KPC_VIR-TC successfully transferred pJ53-KPC_Vir to *K. pneumoniae* ATCC700603 with a conjugation efficiency of (7.75±2.35) × 10^−5^ under meropenem conditions. In the absence of meropenem, J53-KPC_Vir-TC transferred pJ53-KPC_Vir to *K. pneumoniae* ATCC700603 with a conjugation efficiency of (5.74±1.66) × 10^−5^.

### High transmission efficiency and instability of *blaKPC–rmpA2* fusion plasmid during conjugative transfer

Conjugative transfer experiments revealed that the newly formed *blaKPC–rmpA2* fusion plasmid exhibited high transmission efficiency (Fig. 2), but also instability during prolonged propagation (Fig. 5a, b). Over 15 days of serial passage, progressive dissociation of the KPC-virulence fusion plasmid was observed. By day 5, partial dissociation was detected, and by day 14, the plasmid had fully separated into the original resistance and virulence plasmids, with retention rates exceeding 90% in both hosts. Whole-genome sequencing confirmed that dissociation consistently occurred at the original fusion site, facilitating the continued dissemination of the virulence plasmid. The newly formed fusion plasmid displayed high conjugation efficiency between *K. pneumoniae* and *E. coli*. Under meropenem selection pressure, the fusion plasmid transferred efficiently to *K. pneumoniae* ATCC700603, retaining both the KPC and virulence plasmids, with a conjugation efficiency of (7.75±2.35) × 10^−5^. In the absence of meropenem, some transconjugants lost the resistance plasmid, but the dissociated virulence plasmid maintained a high transfer efficiency of (5.74±1.66) × 10^−5^. These results indicate that the fusion plasmid promotes the conjugative transfer of virulence elements, even in the absence of selective pressure.

**Fig. 4.**
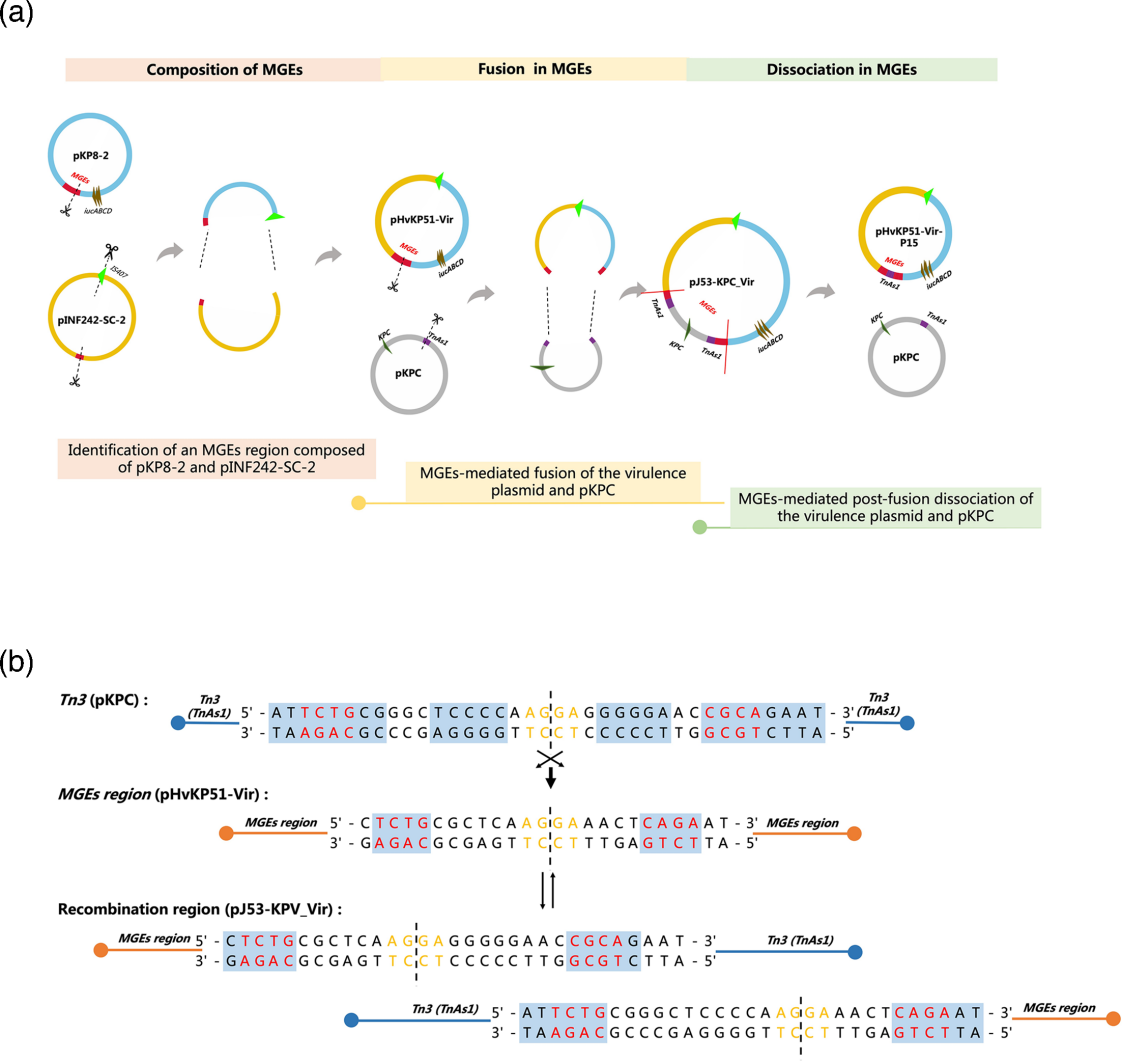
PFGE, S1-PFGE and Southern blot analyses demonstrating plasmid transfer, fusion dissociation and virulence changes in transconjugants in the *G. mellonella* infection model and serum resistance assay. (a) *XbaI* PFGE and S1-PFGE illustrate plasmid transfer, highlighting the new fusion plasmid pJ53-KPC_Vir (yellow), hybrid plasmid pHvKP51-Vir (blue) and pKPC (red). Southern blot confirms that pJ53-KPC_Vir carries both *blaKPC and rmpA2* genes. (b) Stability of the fusion plasmid pJ53-KPC_Vir during serial passages in *E. coli* J53 and *K. pneumoniae* ATCC700603. Dissociation begins after 5 days and is complete by 15 days in both hosts. (c, d) Changes in virulence of *E. coli* J53 transconjugants harbouring pJ53-KPC_Vir (J53-KPC_Vir-TC) and after 15-day passage (J53-KPC_Vir-P15) in the *G. mellonella* infection model and serum resistance assay. Virulence ranking: J53-KPC_Vir-P15>J53-KPC_Vir-TC>J53. (e, f) Virulence changes in *K. pneumoniae* ATCC700603 transconjugants harbouring pHvKP51-Vir (ATCC700603-Vir-TC), pJ53-KPC_Vir (ATCC700603-KPC_Vir-TC) and after passage (ATCC700603-KPC_Vir-P15) in the *G. mellonella* infection model and serum resistance assay. Virulence ranking: ATCC700603-KPC_Vir-P15 ≈ ATCC700603-Vir-TC > ATCC700603-KPC_Vir-TC>ATCC700603. Statistical analysis was performed using the log-rank (Mantel-Cox) test. *P<0.05; ***P<0.001.

### Impact of plasmid fusion on virulence and resistance

**Fig. 5.**
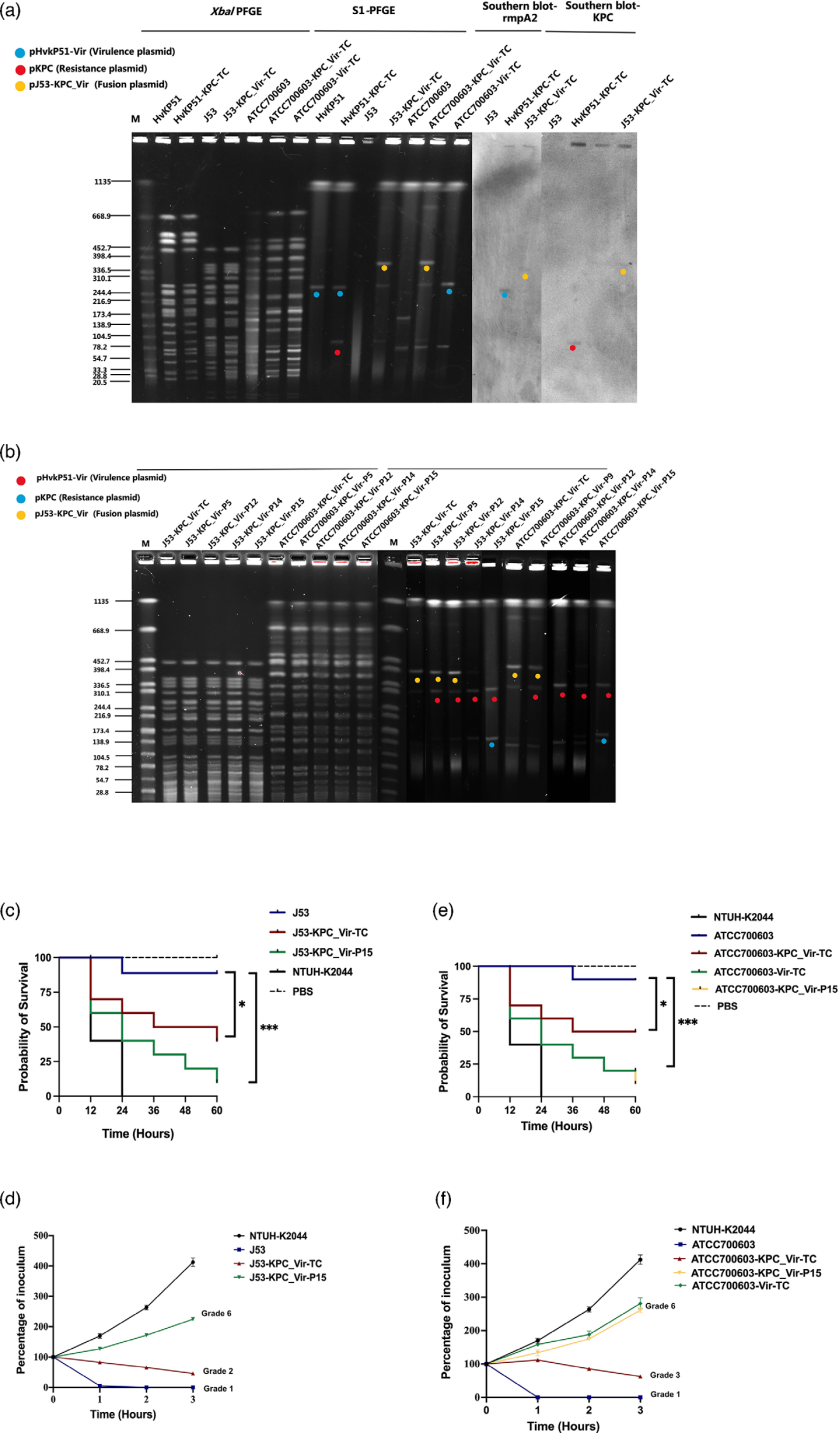
MGE-mediated plasmid recombination and repeat sequences. (a) Schematic of MGEs-mediated plasmid recombination and dissociation. The hybrid plasmid pHvKP51-Vir undergoes two recombination events within a specific MGEs region. (b) The specific recombination site within the MGEs region between the virulence plasmid and pKPC features a unique 8 bp repeat sequence (5′-TCTG-CAGA-3′).

The fusion of KPC and virulence plasmids simultaneously enhanced both virulence and carbapenem resistance in the host strains, though a relative reduction in overall virulence was observed. In virulence assays, the transconjugant J53-KPC_Vir-TC, harbouring the fusion plasmid, exhibited significantly higher virulence compared to the recipient strain J53. Following serial passages, the virulence of J53-KPC_Vir-P15 further increased, surpassing J53-KPC_Vir-TC (Fig. 5c, d). When ATCC700603 was used as the recipient strain, two transconjugants were obtained: ATCC700603-KPC_Vir-TC, which acquired the fusion plasmid pJ53-KPC_Vir, and ATCC700603-Vir-TC, which retained only pHvKP51-Vir. Both transconjugants exhibited significantly elevated virulence compared to ATCC700603, with ATCC700603-Vir-TC and passaged ATCC700603-KPC_Vir-P15 showing higher virulence than ATCC700603-KPC_Vir-TC (Fig. 5e, f). Despite the observed changes in virulence, carbapenem resistance remained stable across all transconjugants, even after multiple passages, underscoring the stability of the resistance elements within the fusion plasmid.

### Whole-genome analysis of fusion plasmid pJ53-KPC_Vir: role of TnAs1 and MGEs in facilitating recombination

Whole-genome sequencing uncovered a complex genetic structure surrounding the KPC gene, featuring diverse MGEs. Following plasmid fusion, the KPC-carrying plasmid acquired an additional Tn3 copy (TnAs1). Detailed analysis identified an 8 bp inverted repeat sequence (5′-TCTG-CAGA-3′) at the TnAs1 insertion site in the virulence plasmid, which likely serves as a stable recombination site enabling plasmid fusion (Fig. 4b).

Analysis of the virulence plasmid revealed that the MGE region had undergone two distinct recombination events (Fig. 4a). The fusion between the virulence plasmid (pHvKP51-Vir) and the KPC plasmid was identified as part of a broader recombination mechanism. This fusion occurred within the same MGE region implicated in prior recombination events between pKP8-2 and pINF242-SC-2. Sequence analysis of this region identified the arrangement IS1-pINF242-2 insertion site-IS407-IS110-pKPC insertion site-IS1, which was shown to facilitate these recombination events. The highly active recombination region within pHvKP51-Vir predisposes it to frequent genetic exchanges at this locus.

## Discussion

In this study, we identified a novel conjugative IncHI1B/repB virulence plasmid, pHvKP51-Vir, in a *K. pneumoniae* ST485-KL5 isolate, which resulted from recombination between plasmids pKP8-2 and pINF242-SC-2. Our *in vitro* conjugation experiments demonstrated that pHvKP51-Vir can merge with pKPC to form the carbapenem-resistant and virulent plasmid pJ53-KPC_Vir in both *K. pneumoniae* and *E. coli*. Subsequent experiments further revealed that this fusion significantly enhanced the dissemination potential of the virulence plasmid. We identified an MGE region that was involved in two recombination events. This MGE region harbours many transposable elements, which likely account for the high recombination activity observed at this locus.

Despite the ability of the fusion plasmid to confer both carbapenem resistance and virulence, it exhibited instability, dissociating back into pHvKP51-Vir and pKPC after 15 days. While the fusion plasmid is transmitted efficiently in *K. pneumoniae* and *E. coli*, it showed slightly reduced virulence compared to the original virulence plasmid. However, following dissociation, the virulence potential increased significantly. The fusion plasmid acts as an intermediate for the high-efficiency co-transfer of virulence and resistance traits. This bacterial evolution, marked by a temporary partial loss of virulence during conjugation, warrants attention, particularly as lost virulence can be regained through subsequent host adaptation. Consistent with previous studies on plasmid adaptation in bacterial strains [39,40], the fusion plasmid, which carries both resistance and virulence genes, appears to reduce the adaptive cost associated with conjugation. The efficient spread of this fusion plasmid may contribute to the clinical prevalence of plasmids conferring both resistance and virulence [41].

We report the fusion of a hybrid virulence plasmid with pKPC during conjugation, a phenomenon that has been underexplored in the context of *K. pneumoniae*. Detailed analysis of the MGEs region identified the sequence IS1-pINF242-2 insertion site-IS407-IS407-IS110-IS110-pKPC insertion site-IS1, which facilitated the observed recombination events.

The emergence of carbapenem-resistant hypervirulent *K. pneumoniae* represents a major challenge for clinical treatment and public health. Our study underscores the potential for virulence and resistance genes to be co-transferred through plasmid fusion, complicating infection control and therapeutic strategies. A deeper understanding of the molecular mechanisms underlying plasmid fusion may lead to the development of novel strategies to prevent the dissemination of these highly resistant and virulent strains. Targeting critical components involved in plasmid fusion, such as MGEs, offers promising avenues for therapeutic intervention.

While our study offers valuable insights into the mechanisms of plasmid fusion, several limitations must be acknowledged. The in *vitro* conjugation experiments may not fully reflect the complexity of plasmid transfer and fusion in natural environments. Moreover, the long-term stability of the fusion plasmid within diverse bacterial populations under varying environmental conditions remains unexplored. Further research is necessary to confirm these findings and investigate additional factors that may influence plasmid fusion and stability.

## Conclusion

In conclusion, our study identifies and characterizes a novel conjugative IncHI1B/repB virulence plasmid, pHvKP51-Vir, in *K. pneumoniae*, capable of fusing with pKPC to form the carbapenem-resistant and virulent plasmid pJ53-KPC_Vir. We propose that this fusion was jointly facilitated by TnAs1 on pKPC and the MGE region on the virulence plasmid. The resulting fusion plasmid, although capable of conferring both carbapenem resistance and enhanced virulence, exhibits temporal instability. These findings offer novel insights into the molecular mechanisms underlying plasmid fusion and emphasize the critical roles of mobile genetic elements in the evolution of hypervirulent and antibiotic-resistant bacterial strains. Advancing knowledge in this domain will be vital in addressing the clinical and public health challenges posed by carbapenem-resistant hypervirulent *K. pneumoniae*.

## Supplementary material

10.1099/mgen.0.001534 Uncited Supplementary Material 1.

## Abbreviations

CR-HvKP: carbapenem-resistant hypervirulent *Klebsiella pneumoniae*
IS: insertion sequence
LB: lysogeny broth
MGEs: mobile genetic elements
pKPC: KPC-type resistance plasmid
